# Visualizing Neural Pathways Affected by Alcohol in Animals

**Published:** 1995

**Authors:** David Lyons, Linda J. Porrino, Susanne Hiller-Sturmhöfel

**Affiliations:** David Lyons, Ph.D., is a research associate and Linda J. Porrino, Ph.D., is an associate professor in the Department of Physiology and Pharmacology at the Bowman Gray School of Medicine, Wake Forest University, Winston-Salem, North Carolina. Susanne Hiller-Sturmhöfel, Ph.D., is a science editor of Alcohol Health & Research World

**Keywords:** neuron, animal model, AODE (alcohol and other drug effects), blood circulation, protein synthesis, enzymes, dose response relationship, glucose, radionuclide imaging, positron emission tomography, magnetic resonance imaging

## Abstract

Alcohol has many short- and long-term effects on functional units of nerve cells, or neural pathways, in the brain. Imaging studies in laboratory animals, particularly studies using autoradiographic detection methods, allow researchers to analyze the activities of these pathways as well as alcohol’s effects on them. Commonly used techniques measure blood flow, glucose utilization, protein synthesis, and the activity of the enzyme cytochrome oxidase throughout the animal’s brain. Using these approaches, researchers have investigated alcohol’s effects both in animals that receive alcohol for the first time and in animals with a history of alcohol exposure. These studies demonstrate that different alcohol doses affect different neural pathways and that the affected brain areas also vary depending on the time that has elapsed since alcohol ingestion. Moreover, chronic alcohol use can markedly alter the baseline activity of certain neural pathways, even in animals that are not alcohol dependent.

Alcohol is a pharmacologically complex agent that acts throughout the body via several different biological mechanisms (Nutt and Peters 1994). The overall effect depends on the amount of alcohol consumed, the time elapsed following ingestion, the rates of absorption and elimination (i.e., how fast the alcohol is absorbed into both the blood and the brain, and how fast the alcohol is metabolized in the body), and the response of particular tissues. In turn, metabolism and tissue response are influenced by certain characteristics of the drinker, such as age, gender, body weight, nutritional and general health status, and drinking history. Alcohol’s effects on the brain also may be influenced by a person’s lifestyle, the social context of alcohol consumption, and various psychological variables. Studies have shown, for example, that social drinkers consume more alcohol and report increased pleasure when they drink with others than when they drink alone (Doty and de Wit 1995). This multitude of factors, some of which cannot be easily controlled in clinical studies, makes it difficult to assess alcohol’s effects on the human brain. Therefore, many researchers use laboratory animals as models to study alcohol’s effects, because animal studies allow scientists to control their subjects’ histories and genetic backgrounds as well as the environmental conditions under which the experiments take place.

Unlike other organs, the brain comprises a myriad of anatomical components, each possessing a different level of organization and function. This complex organization is accomplished by linking together cells from different brain areas into functional units called neural pathways, or neuroanatomical circuits. The existence of these circuits implies that only a subset of brain cells, or neurons, needs to respond to any given stimulus. For example, an animal’s first exposure to alcohol may alter the activity of particular pathways. After several weeks of alcohol exposure, a different set of pathways may be affected, owing to the animal’s developed tolerance to the alcohol stimulus and to the corresponding physiological changes in the neurons. Researchers are attempting to identify the neural pathways affected by alcohol and to quantify the alcohol-induced changes in the living organism. A better understanding of the initial and residual effects of alcohol in the brain will help to better explain the causes of performance deficits in alcoholics and possibly aid in the development of new treatment approaches.

Alcohol is only one of several commonly abused drugs. Other drugs, such as cocaine and heroin, also produce dramatic changes in brain activity. An important question addressed by alcohol studies in animals is whether all abused drugs affect a common neural pathway, sometimes referred to as the brain “reward system.” If such a common pathway exists, similar therapies might successfully treat various drug addictions. Conversely, differences between the mechanisms of action of various drugs could explain why some treatments are effective for one class of abused drugs and not for others.

Brain imaging enables researchers to investigate these issues in animal models. This article describes several imaging techniques used to analyze alcohol’s effects on the brain’s pathways and presents the results of studies that used these imaging methods to investigate the consequences of acute and chronic alcohol consumption.

## Imaging Using Autoradiography

Autoradiography is the process of detecting the distribution of a radioactive substance in a nonliving sample, such as a tissue slice, by exposing the sample to x-ray film. The radioactive substance, called a tracer, mimics a compound naturally found in the tissue of interest and contains at least one weakly radioactive atom. In structural imaging approaches that measure the presence and quantity of cellular components, the tracer may be applied directly to the tissue slice. (For more information on such approaches, see sidebar, p. 302.) Conversely, when autoradiography is used to assess the activity or function of a tissue, the tracer often is administered to the living subject. Thus, these latter studies can be conducted in the awake, fully reactive subject. This aspect is crucial, because the environmental context has a profound impact on how psychoactive substances, such as alcohol, act on the brain. However, because researchers also require actual tissue slices to detect the tracer, these techniques can only be conducted on animals. Additional advantages of using animals, rather than humans, in this research include better control of experimental variables, lower costs, and shorter experiment durations.

**Structural Imaging Techniques**

Structural imaging techniques detect the quantity of particular cell components, such as the receptors to which neurochemicals can bind. However, these techniques generally do not allow conclusions to be drawn about the resultant changes in cell function when these structural components are modified.

***Receptor-Binding Autoradiography***

***What Is Visualized?*** Docking molecules (i.e., receptors) reside in the cell surface to interact with signaling molecules, such as neurotransmitters or hormones. Radiolabeled tracer molecules mimic natural neurotransmitters or hormones, attaching themselves to the receptors much like a key in a lock.

***How Is It Done?*** Very thin tissue sections are incubated in a liquid medium with the tracer, allowing the tracer to bind. The tissue is then dried and exposed to x-ray film. The radioactive substance produces black spots on the film. The resulting image indicates where the tracer has accumulated.

***What Is Gained?*** By examining tissue throughout the brains of experimental animals, researchers can determine the regional distribution of receptors, which provides important clues to brain function and how alcohol affects it.

***Example of the Technique.*** The brains of mice repeatedly exposed to alcohol throughout their lives exhibited increased activity of the receptor that binds the neurotransmitter *N*-methyl-D-aspartate (NMDA). The length of time needed for this binding paralleled the time-course for the increased susceptibility to seizures during alcohol withdrawal (Gulya et al. 1991). These data provide evidence that the alcohol-induced changes in the NMDA receptor may be responsible for particular withdrawal symptoms.

***In Situ Hybridization Histochemistry***

***What Is Visualized?*** Changes in the production of certain proteins generally are preceded by changes in the amount of messenger ribonucleic acid (mRNA) for those proteins (mRNA is an intermediary molecule formed during the process of converting genetic information encoded in the DNA into a protein product). Radio-labeled DNA or RNA tracer molecules can attach themselves to the mRNA.

***How Is It Done?*** As with receptor-binding studies, very thin tissue sections are incubated in a liquid medium with the tracer, allowing the tracer and mRNA to join together. The tissue is then dried and exposed to x-ray film, and the resulting image is analyzed.

***What Is Gained?*** Although all cells in an organism (with the exception of reproductive cells) contain the same amount of DNA, the amounts of mRNA—which instructs the cell to produce specific proteins (e.g., receptors)—vary in response to certain environmental conditions, such as alcohol exposure. The regional distribution of changes in mRNA content provides more evidence of how the brain responds to alcohol.

***Example of the Technique.*** In mice chronically exposed to alcohol, mRNA levels for prodynorphin, a precursor of opioids (i.e., natural substances with opiumlike effects), increased in several brain areas, including the hypothalamus. Conversely, mRNA levels for vasopressin, another neurotransmitter found in the hypothalamus, decreased (Gulya et al. 1993). This demonstrates that alcohol has complex effects on different mRNA’s.

—David Lyons, Linda J. Porrino, and Susanne Hiller-Sturmhöfel

ReferencesGulyaKGrantKAValveriusPHoffmanPLTabakoffBBrain regional specificity and time-course of changes in the NMDA receptor-ionophore complex during ethanol withdrawalBrain Research54712913419911830510GulyaKOrphanaAKSikelaJMHoffmanPLProdynorphin and vasopressin mRNA levels are differentially affected by chronic ethanol ingestion in the mouseMolecular Brain Research20181993825517010.1016/0169-328x(93)90105-x

The primary technical advantage of these procedures over similar studies in humans is the excellent ability of autoradiography to distinguish between adjacent structures (i.e., spatial resolution). Improved spatial resolution also distinguishes autoradiography from the strictly biochemical methods from which it was derived. These biochemical methods usually require gross dissection of tissue, which is then homogenized, or pureed, for analysis.

Because autoradiography enables researchers to experiment on an intact tissue slice, the results are in keeping with the brain’s highly complex anatomy. Some techniques can even detect changes at the level of single nerve cells, or neurons. Most autoradiographic studies also can examine the entire brain rather than a single piece of tissue, thereby revealing activity patterns throughout the brain. It is these patterns that indicate the activity of particular neural circuits. Using this neuroscientific approach, conditions can be identified under which specific neural systems are activated (referred to as a systems approach).

## Functional Imaging Techniques

Several functional imaging techniques have been used in conjunction with animal models to study the effects that alcohol and other drugs exert on the brain. Four of these approaches—regional cerebral blood flow (RCBF), local cerebral glucose utilization (LCGU), local cerebral protein synthesis (LCPS_leu_), and cytochrome oxidase activity—are discussed in the following sections.

### Regional Cerebral Blood Flow

To function adequately, the brain must be supplied continuously with nutrients. In contrast to other organs, the brain cannot store sufficient nutrients and therefore depends on a constant supply through the blood. Brain regions that are active at any given time require more nutrients than inactive brain regions and thus have a higher rate of blood flow. These characteristics form the basis for measuring RCBF (Sokoloff 1986).

This imaging approach capitalizes on the fact that anything injected into the bloodstream is distributed rapidly to the various tissues throughout the body according to each tissue’s need for blood and its supply of nutrients and oxygen. Thus, to determine RCBF, researchers inject a radioactive substance into the animal’s bloodstream. Several substances can be used as long as they are chemically inactive, dissolve rapidly in the blood, are not quickly metabolized, and can cross the blood-brain barrier.^1^ Shortly after injection (usually about 1 minute), the animal is euthanized, and its brain is quickly frozen for later analysis. Very thin brain slices are collected and exposed to x-ray film. The brain areas that were active, and thus had high rates of blood flow, will have accumulated more radioactive material, visible as dark areas on the film, than the less active regions. The advantage of RCBF over other functional imaging methods is that researchers determine blood flow during a short time period, allowing them to investigate highly dynamic brain processes that change rapidly over time.

The RCBF measurements can be used to examine brain function under different experimental conditions. For example, to investigate the potential mechanisms leading to developmental abnormalities after neonatal alcohol exposure, researchers studied newborn piglets that were administered alcohol. The study found that even when alcohol led to a 30-percent change in the piglets’ arterial blood pressure, the animals’ RCBF remained constant, suggesting that any developmental abnormalities were likely not attributable to insufficient blood supply to the brain (MacIntyre et al. 1987).

### Local Cerebral Glucose Utilization

Like RCBF, the assessment of LCGU rates relies on the unique properties of brain tissues. The rationale for using this method, which was developed in the 1970’s (Sokoloff 1986), is that approximately 80 percent of the energy neurons consume is used to transmit nerve signals (i.e., the mechanism by which information is transferred in the brain) and only about 20 percent of the energy maintains the cells’ baseline metabolism (Kurumaji et al. 1993). Furthermore, glucose, a sugar which is absorbed from the bloodstream, is virtually the brain’s only energy source. Therefore, a rise in glucose utilization in a particular brain area indicates increased neural activity in that region.

Researchers measure glucose utilization by intravenously injecting animals with a radioactively labeled substance, 2-[^14^C] deoxyglucose (2–DG). This compound is chemically similar to glucose and, thus, is readily transported into neurons. The investigator obtains sections of the animal’s brain for analysis 45 minutes after injecting the 2–DG. Because the tracer is administered in one short intravenous infusion, most of the 2–DG is quickly absorbed and trapped in the brain. In addition, 2–DG cannot be metabolized past the initial stage of energy production; therefore, it remains trapped in the cells. Consequently, the effective imaging window is actually shorter than the experimental period, and the method primarily visualizes events occurring during the first 10 to 15 minutes of the experiment. Allowing the tracer to clear from the blood for the full 45 minutes, however, makes the calculation of glucose utilization much easier and more accurate.

As with RCBF, the researcher sections the animal’s brain into thin slices and exposes them to x-ray film to detect the amount of radioactivity in different brain regions. Regions with high neural activity accumulate greater amounts of 2–DG and appear as dark areas on the x-ray film. The advantages of LCGU over RCBF are the excellent spatial resolution of the images, the reliability of the measurements, and the well-understood link between cerebral metabolism (i.e., glucose utilization) and cellular activity. These advantages are chiefly the result of the measurement of the biochemical process of glucose metabolism, which is more precise than blood flow analysis and is tightly coupled with neural activity.

### Local Cerebral Protein Synthesis

Using the same basic principles and approach as for estimating LCGU, Sokoloff (1986) also developed a technique to determine LCPS_leu_. Dramatic alterations in the amount of protein in a brain region suggest a fundamental change in function, because proteins compose much of the basic machinery necessary for cellular function. All proteins are made up of amino acids linked together in a chain, and most proteins contain the amino acid leucine. Consequently, researchers assess LCPS_leu_ by determining the rate at which radiolabeled leucine is incorporated into proteins in the brain. Unlike RCBF or LCGU, however, LCPS_leu_ does not necessarily correlate with nerve cell function (i.e., neural firing rates), but is instead a general measure of the formation of new proteins. One important disadvantage of LCPS_leu_, however, is its lack of selectivity: It determines changes in the total protein level rather than measures of specific proteins.

Although LCPS_leu_ analysis has not been applied to alcohol research, it has been used to study cocaine’s impact on the brain (Orzi et al. 1995). A single (i.e., acute) dose of cocaine reduced LCPS_leu_ throughout the brain, especially in the cortex, basal ganglia, and amygdala.^2^ Tolerance to these acute effects developed after 1 week of daily injections; however, a single cocaine injection subsequent to chronic treatment and an additional week of no treatment revealed a pattern of LCPS_leu_ that differed from the acute pattern. These data suggest that a long-lasting change in the brain’s response to cocaine occurs after prolonged exposure. Similar experiments also may prove useful for studying the long-term consequences of alcohol exposure.

### Cytochrome Oxidase Activity

Cytochrome oxidase is the last enzyme in the electron transport chain, a key component of the cellular process by which oxygen is used for energy production (i.e., aerobic respiration). Mammals’ brains principally rely on aerobic respiration to meet their energy needs. Consequently, the activity levels of cytochrome oxidase are closely linked to neural activity. Several researchers have measured cytochrome oxidase activity histochemically in animal tissue to identify functional activity patterns (Wong-Riley 1989). Other researchers have attempted to develop a quantitative histochemical method of cytochrome oxidase measurements (Gonzalez-Lima and Jones 1994).

Alterations in cytochrome oxidase activity require the production or degradation of the enzyme, both of which occur within hours or days. Conversely, LCGU or RCBF may change within minutes. Thus, although these three techniques are linked to neuronal activity, changes in cytochrome oxidase activity reflect longer term alterations. Thus, researchers may find this technique particularly useful in studies in which brain regions take on new functions—for example, during early development, in response to trauma, and, potentially, after chronic alcohol exposure. To date, however, no studies have employed this method to examine the effects of alcohol and other abused drugs.

## Detecting Alcohol’s Effects on Neuroanatomical Circuits

Scientists have used imaging techniques assessing cellular structure and function to examine the complex effects of alcohol and other drugs on the brain under various experimental conditions. Functional imaging techniques have proved to be especially useful, because it is often extremely difficult to predict which brain structures alcohol will affect. The following sections describe some of the findings from imaging studies that identified alcohol’s effects on neuroanatomical circuits in animals.

### Acute Alcohol Effects in Animals Without Histories of Alcohol Exposure

#### Dose-Dependent Alcohol Effects

A variety of studies have described the biphasic effects of acute alcohol administration (Pohorecky 1977). Animal studies found that low alcohol doses commonly had stimulatory effects, leading to increased movement and exploration of the testing environment, whereas higher doses tended to suppress behavior or induce sedation. Similarly, researchers found differences in the release of some neurotransmitters (e.g., dopamine) after low and high alcohol doses (Engel and Lilijequist 1983). Few studies have examined in detail the brain regions affected by different alcohol doses (Eckardt et al. 1988; Williams-Hemby and Porrino 1994). Until recently, it was unclear whether the changes induced by low and high alcohol doses affected the same neural circuits differently or whether they affected different brain systems altogether.

Williams-Hemby and Porrino (1994) addressed this issue by measuring LCGU’s in alcohol-naive rats (i.e., animals that previously had not been exposed to alcohol) after the animals received one of two different alcohol doses: a low dose corresponding to approximately one drink in a human or a higher dose corresponding to about four drinks. When the animals’ blood alcohol concentrations (BAC’s) reached their peak, the researchers determined the LCGU’s in 54 brain regions.

**Figure 1:**
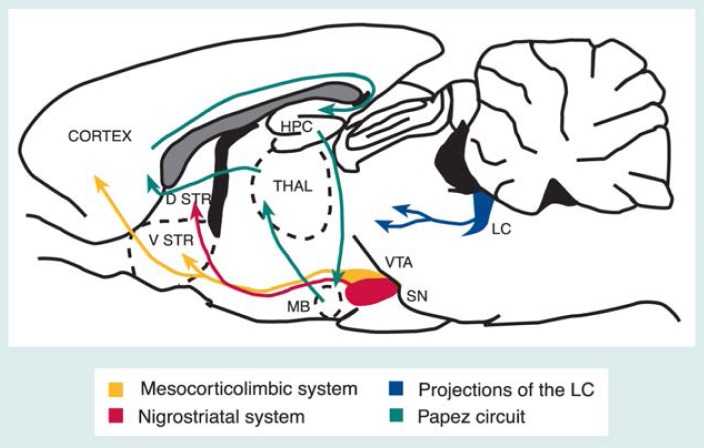
Schematic diagram of a lengthwise cros-section of the rat brain (i.e., separating the two halves of the brain) illustrating some of the neural circuits affected by alcohol intake. D STR = dorsal striatum; HPC = hippocampus; LC = locus ceruleus; MB = mammillary bodies; SN = substantia nigra; THAL = thalamus; V STR = ventral striatum, which comprises the nucleus accumbens and the olfactory tubercle; VTA = ventral tegmental area.

This study found that both alcohol doses altered neural activity in several brain areas but the patterns of change differed between the doses (Williams-Hemby and Porrino 1994). The low alcohol dose increased neural activity, particularly in brain structures using the neurotransmitter dopamine that have been associated with reward and positive reinforcement (i.e., the mesocorticolimbic system) and motor activity (i.e., the nigrostriatal system) (see figure 1). Conversely, the higher alcohol dose distinctly changed activities in structures related to the processing of sensory and motor information (i.e., the thalamus), memory and behavioral functions (i.e., the hippocampus), and arousal (i.e., the locus coeruleus) (figure 1).

These findings allowed two major conclusions. First, different alcohol doses distinctly affected different brain regions, rather than acted on a single system in a dose-dependent manner. Second, the brain structures in which the researchers detected changes in functional activity were consistent with the observed, alcohol-induced behavioral changes. The low alcohol dose led to a widespread increase in brain activity in areas related to alcohol’s arousing and rewarding effects on behavior, whereas the higher alcohol dose suppressed brain function and had more suppressive and sedating effects on behavior.

#### Time-Dependent Alcohol Effects

Alcohol’s effects on behavior and brain functioning depend not only on the amount of alcohol consumed but also on the time that has elapsed since the alcohol was ingested. Behavioral studies in both animals and humans indicate that soon after alcohol consumption, when the BAC is rising or at its peak, alcohol’s activating and rewarding effects prevail (Lukas et al. 1991; Lewis and June 1990; Risinger and Cunningham 1992). Later, when the BAC is falling, alcohol’s depressive and sedating effects predominate.

To investigate whether functional changes in brain activity reflect these behavioral observations, Porrino (1992) analyzed the effects of a moderate alcohol dose (0.5 gram per kilogram of body weight [g/kg]) on the LCGU of rats at both 10 and 40 minutes after alcohol administration. The researchers chose these time points so that the LCGU measurement coincided with the BAC’s rising (i.e., at 10 minutes) and falling (i.e., at 40 minutes). At both time points, the LCGU had increased in several brain regions, including structures within the mesocorticolimbic and nigrostriatal systems. However, in one of the mesocorticolimbic system’s structures, the olfactory tubercle, the LCGU had increased at the earlier time point but returned to normal by the later time point. As previously noted, the mesocorticolimbic system has been associated with reward and reinforcement. Given that alcohol’s rewarding aspects appear to predominate soon after alcohol ingestion, and that the region containing the olfactory tubercle is involved in altering mood, alcohol’s selective effects on the olfactory tubercle suggest a unique role for this structure in processing alcohol’s reinforcing effects.

### Acute Alcohol Effects in Animals With Histories of Alcohol Exposure

Several studies have used the 2–DG method to analyze alcohol’s effects on LCGU in animals after prolonged alcohol exposure (Eckardt et al. 1992; Grunwald et al. 1992). In these studies, chronic alcohol ingestion decreased brain activity in the limbic system, which encompasses parts of the hippocampus and other structures related to memory and emotions; in the striatum, which is related to motor activity and cognition; and in the cerebellum, which also controls aspects of fine motor control. These functional changes share some similarities, with changes in brain activity resulting from a single alcohol dose in otherwise alcohol-naive animals (described previously). However, in several brain structures of chronically treated animals, alcohol’s effects were markedly diminished or completely absent. This finding indicates that in some brain structures, the response to alcohol becomes weaker over time (i.e., tolerance develops). This interpretation is consistent with behavioral studies in both animals and humans which demonstrate that although tolerance to some of alcohol’s effects develops after chronic alcohol exposure, alcohol’s deleterious effects on functions such as memory and cognitive performance persist or even worsen over time (Nathan 1990; Arendt 1994).

In contrast to human alcoholics, who consume alcohol over many years, the animals in many of these studies received high alcohol doses over relatively short periods of time (i.e., within a few days). To address this issue, Williams-Hemby and colleagues (in press) used an animal model that more closely resembled human drinking behavior by training rats to consume relatively large amounts of alcohol voluntarily for several months without actually becoming alcohol dependent. On the test day, the researchers assessed functional activity immediately after the animals had ingested alcohol and compared the results with the brain activity of control animals that had received only water throughout the experiment. The alcohol-drinking animals showed a highly distinctive pattern of change in regional brain activity compared with the control animals: The brain activity of the alcohol-drinking animals was significantly reduced in structures belonging to the so-called Papez circuit, a limbic system consisting of parts of the hippocampus, sections of the thalamus, and the mammillary bodies. Simultaneously, brain activity increased in portions of the mesocorticolimbic system and in the locus coeruleus, two brain systems that have been associated with mood, reinforcement, and arousal.

The consequences of chronic alcohol ingestion on brain functioning can be determined not only in the presence but also in the absence of acute alcohol exposure, allowing researchers to assess changes in the baseline functioning of the brain that have been induced by long-term alcohol use. Scientists have used two experimental designs to determine these changes. Some studies were performed on alcohol-dependent animals that did not receive alcohol on the test day and, consequently, developed withdrawal symptoms (Eckardt et al. 1992). Other studies examined the animals under conditions in which acute withdrawal symptoms did not develop (Williams-Hemby et al. in press).

Compared with control animals that never received alcohol, animals suffering from acute withdrawal symptoms exhibited dramatically increased functional activity in many brain areas, including regions associated with motor and auditory functions (Campbell et al. 1982; Eckardt et al. 1992). These findings are consistent with results from other studies that detected hyperactivity in the central nervous system during withdrawal episodes. Williams-Hemby and colleagues (in press) observed similar effects when they studied rats that voluntarily consumed large amounts of alcohol but were not alcohol dependent and, therefore, did not experience acute withdrawal when alcohol was withheld. Again, functional activity throughout the brain increased in these animals, compared with control animals that never received alcohol. Thus, marked changes in brain functioning after chronic alcohol use occur even in the absence of acute withdrawal symptoms.

## Conclusions

Studies using functional imaging techniques have shown that alcohol does not always affect the same brain regions. The constellation of brain structures exhibiting changes in functional activity varies depending on the alcohol dose, the time elapsed following alcohol consumption, and the individual’s drinking history. The studies described here allow the following conclusions regarding the patterns of brain-functioning changes and their consequences:

- After one-time consumption of low alcohol doses and when the BAC is rising, functional activity increases primarily in the mesocorticolimbic system, portions of which constitute the “reward system.” Because alcohol affects this system in the same way as other abused drugs, a common pathway may exist for drug-induced mood changes.
- After consumption of higher alcohol doses and when the BAC is falling, functional activity decreases in several brain structures, including parts of the auditory system, the nigrostriatal motor system, the hippocampus, the thalamus, and the locus coeruleus. These brain structures have been associated with cognitive and memory performance, sensory and motor function, and arousal. The functional decrease in these areas is consistent with reduced performance of these functions in other analyses.
- Chronic alcohol consumption reduces the responsiveness of some brain areas to an acute alcohol dose, indicating tolerance development in these regions. Other brain circuits, however, remain sensitive to alcohol’s effects even after repeated alcohol exposure. These brain circuits primarily include the hippocampus, which is related to memory and other behavioral functions, as well as other limbic structures. These findings are consistent with behavioral observations that alcohol-induced impairment of cognitive and memory functions persists or even intensifies after long-term alcohol use.
- Chronic alcohol consumption alters the baseline functional activity of many brain regions. Animals with drinking histories showed dramatic increases in functional activity throughout the brain when alcohol was withheld, even in the absence of overt behavioral withdrawal signs. These changes may represent the existence of a mechanism that compensates for chronic alcohol exposure.

Functional autoradiographic imaging techniques have enabled researchers to study in great detail alcohol’s effects on individual brain structures and their circuits. These imaging methods can provide a sensitive means to investigate the development of physical dependence and withdrawal symptoms as well as offer a method for examining treatment effectiveness.

The studies described here have focused on alcohol’s effects in animal models, rather than in humans, not only for technical reasons but because animal models enable investigators to control experimental variables. New imaging technologies, such as positron emission tomography (PET), however, now allow similar studies to be conducted in humans and other primates. One advantage of these new imaging methods is that subjects can be studied repeatedly; thus, changes occurring over time can be examined on an individual basis. PET and similar technologies still provide only relatively limited spatial resolution; however, the prospects for improved functional imaging in humans are good. New PET scanners with improved resolution soon will be available, and magnetic resonance imaging technology, which has excellent spatial resolution, is making great strides in the functional imaging arena. With a judicious combination of animal and human experiments, researchers can most effectively study the consequences of alcohol abuse and dependence.

## Footnotes

1 The term “blood-brain barrier” refers to a property of blood vessels in the brain that allows the exchange of only certain molecules between the blood and the brain.

2 For a description of the location and function of these and other brain regions and for a definition of this and other technical terms used in this article, see figures and central glossary, pp. 293–295.
